# Veratrum Viride

**Published:** 1880-08-20

**Authors:** W. C. Norwood


### VERATRUM VIRIDE.

BY W. C. NORWOOD, M.D., S. C.

The primary and direct effect is to control and regulate the action of the heart and arteries in all febrile and inflammatory diseases, be their name and character what they may. It is not for us to attempt to limit the application of a remedy endowed with such powers in the treatment of disease. We state further, that it meets many, if not all, of the prominent indications in the treatment of disease. It produces the very opposite effects, and therefore becomes the counter agent of all diseases. It certainly and in all cases renders a frequent and weak pulse slow, full and distinct; a flushed, hot and dry surface more or less pale, cool and moist.

This it does without exciting the least nausea or vomiting, by giving to an adult male five drops, and increasing the dose one drop every portion given at the end of every third hour, till the pulse is reduced to sixty-five beats per minute. When the pulse is reduced to this number, do not increase the drops. The pulse may be kept at sixty-five beats indefinitely, without causing the slightest nausea or vomiting. The pulse may be reduced to forty-five, forty, and even thirty-five beats, by a very gradual increase of the dose, without exciting the least nausea. But you cannot keep it reduced any length of time to either of these numbers, even if you do not increase the dose, without causing the most intense nausea and vomiting, profuse perspiration, coolness, or even icy coldness of the surface, great paleness, or even palor, and in nervous or hysterical persons a sensation of strangling, difficulty of breathing, and a feeling of suffocation, a perfect paroxysm of globus hystericus, which alarms the patient, friends, and even physician if not familiar with the effect of the remedy. We stated these in one of the five first articles we published, that many never read. You cannot reduce the pulse below the last numbers named by any other remedy without inducing all of the above named apparently alarming effects. We know of what we affirm, when we say apparently, for there is no real danger, as we shall abundantly show.

The blood being furnished to the brain so slowly (for the blood is the life thereof), all of the vital functions suffer, and the peculiarly disagreeable effects follow. If the effects of veratrum viride in doses of from 30 to 60 drops, or even in doses of two ounces of the tincture, or more, were in proportion, death would speedily follow. But vomiting rapidly follows the large portions, and the supply of blood to the brain is diminished temporarily, and all of the effects soon pass off, whether aided or unassisted by morphine and brandy.

To illustrate: The effects of bleeding from a large orifice in erect position, *ad deliquum animi* will soon pass off; but to bleed *ad deliquum* from a large orifice in the horizontal position will be recovered from much slower, on acconnt of the deliquum being brought on much slower, and the loss of blood being much greater.

In the case of veratrum viride the blood is all in the system in both cases; but where the pulse is gradually rendered slower by a gradual and slow increase of the drops, the slow return and less decarbonized blood to the brain renders the effects of the veratrum more intense than the large dose of veratrum, for in it the supply of blood is but temporary by lessening, and the blood is also much less carbonized. But in the large loss of blood from bleeding the system sustains greater and more permanent injury.

It is the only agent that will render the number of the pulse slower in health than natural, and not diminish its fullness and strength. It never renders a pulse weak in health or disease except when given in doses sufficiently large to nauseate and vomit. We have been using veratrum viride thirty-six years. We tested its effects carefully on our own person, and watched closely and with interest its peculiar effects on the person of others. After this long use and experience we can state beyond a peradventure that it is destitute of all poisonous effects in any dose, however large or small, in which it has ever been given or ever been taken, either by mistake or design. On the highest testimony it can be established that it has been taken in over two ounces at a single dose, and no ultimate ill effects followed. Prof. Percy, of New York, wrote a learned essay on veratrum viride.

He was awarded a gold medal by the United States Medical Society. He reports many cases in which from two to four ounces were taken, and in all his experience he never knew of an authenticated case of death following its use.

N. O. Pearson, M.D., gives a case of poisoning from veratrum viride, but was cured by opium being given in a most prodigal manner.

Prof. Percy remarks: "We think our friend (Pearson) has erred in the deductions he has drawn, and as he gets better acquainted with the therapeutic effects of his remedy, he will himself acknowledge so. Had he not administered opium his patient would have recovered quite as well, as every one who has used the veratrum to any extent will assure him. We have several times induced just such a state, and purposely kept our patient at that point for over coming his disease," nor have we felt the least alarm.

Dr. F. F. Gary, a prominent physician in this place, had a son of about twenty years attacked with chorea. The disease was the most rapid and intense we ever witnessed. In less than fifteen days it was all that two adults could do to prevent his sustaining injury from the intensity of the convulsions. To show that we do not over state the case, two brothers, absent from home, were telegraphed to hasten home, and one to bring to his assistance another prominent doctor. Although long since retired from practice, we were asked to see him. He was put on the use of veratrum viride. We remarked to the doctor that we had never known a case that did not yield to a full dose in less than two hours. The Doctor, with much ado, had dropped out twelve drops. We remarked, "just tip out three drops more," which he did; in less than one hour the patient vomited freely, and every convulsion arrested; and when the brothers that had been telegraphed for reached their father's with the physician, the patient was perfectly calm, and his muscles were undisturbed by the slightest twitch. The sons were lawyers, and men of sense. One of them remarked to the writer, "It is a perfect triumph; you have immortalized yourself; father was afraid of it."

H. G. Barrows, M.D., of Boston (Mass.), personally unacquainted: "I feel a strong desire to add my testimony in favor of the agent (veratrum viride). In cases of fever, and especially in children, I could not do without it. It has wrought wonders in my experience. The unpleasant effects would have occasioned me no anxiety, as these effects are easily controlled."

We do not rest our case on our own experience and testimony as to the safety and efficiency of our remedy, for we are a deeply interested party. But those that sustain us and bear witness to its safety, are professors and teachers that stand high in their profession; men of honest purpose and integrity, and would scorn to state that a remedy was safe that was poisonous and dangerous, and thereby mislead men of less learning and talent in their profession. If the testimony given is not worthy of confidence and reliable, there is nothing human or short of a miracle that will convince.—*Southern Clinic.*

[The dangers of veratrum have doubtless been greatly exaggerated, and its virtues scarcely appreciated; yet the profession will make allowance for the enthusiasm of Dr. Norwood touching an agent with the history of which his name must be ever connected, and will scarcely risk the heroic doses advised when its good results may be obtained with smaller doses and a more cautious use of the remedy.—EDITOR of *Record.*]

